# Phlyctenular Ophthalmia

**Published:** 1903-01-24

**Authors:** 


### PHLYCTENULAR OPHTHALMIA.

In discussing the treatment of phlyctenular ophthalmia, Mr. Devereux Marshall[1] says that if we examine these patients critically we shall almost invariably find that they are suffering from eczema about the nose, face, or mouth, and sometimes also on other parts of the body. In fact, we shall not be far wrong if we look upon the disease (as its name implies) as a development of eczema upon the conjunctiva itself. For its successful treatment one must study the morbid processes which accompany it, and remember that it occurs far more in underfed and badly-nourished children than in any other class of the community. Its most distressing symptom is photophobia, and its most serious result is impairment of the transparency of the cornea. In regard to the administration of iron, which is so commonly prescribed, Mr. Marshall is convinced that this frequently leads to the condition being much prolonged, the irritability of the eye being aggravated by its too early employment. It is useful enough in the later periods, but the early stages are far better treated with small doses of mercury internally, as by the following powder: grey powder, 2 grains; powdered belladonna leaves, 1 grain; and sugar of milk, 2 grains. Half of such a powder may be given twice a day. In addition atropine is essential, and as a rule drops containing 3 grains to the ounce are to be preferred, although if more convenient atropine ointment of a similar strength may be employed instead. The eye should also be bathed frequently with boracic lotion. Later on an ointment containing 2 to 8 grains of the yellow oxide of mercury in an ounce of vaseline is useful, but in the acute stage it is too irritating. When the eye is sufficiently quiet to allow this ointment to be used it is sometimes convenient to use a little atropine with it (2 grains to the ounce), and at this stage also iron comes in very well. One of the chief causes of the continuance of this symptom is the development of fissures and excoriations at the outer canthus, and if they can be cured half the battle is over. If these fissures be stretched and made to bleed on the child's opening his eyes, a great deal of good is likely to accrue. By far the best treatment is to give an anæsthetic and then to stretch the lids as widely open as possible. This will cause bleeding, but after it has ceased the lesions will heal. In order to make more sure of this result the fissures may with great advantage be painted with a solution of nitrate of silver (5 to 10 grains to the ounce), the same being done also to the lids and to any phlyctenular ulcers which may be present. An old-fashioned method of gaining the same end is to hold the child's face in water till he commences to struggle; in the process the eyes are widely opened under water, and great good results. This method may seem a rough one, but it is astonishing how quickly children get used to it and cease to mind it in the least. The administration of an anæsthetic is, however, far more effectual, and is open to less objection, as the condition present can be seen and treated. In conclusion, a protest must be raised against the use of cocaine when ulceration of the cornea is present or feared. There is nothing to be said against its employment by the surgeon to aid in his examination; but if it is given to the patient to use, it is sure to be applied too frequently, and thus will destroy the epithelium and favour perforation.

[1] Practitioner, January.

